# Observations on Ann Fooks's Case

**Published:** 1814-02

**Authors:** 


*For the Medical and Physical Journal.*

### *Observations on Ann Fooks's Case*; *by* Mr. Gibbon.

THE controversy respecting Ann Fooks may well be said to assume an extraordinary complexion, when the Rev. J. S. Grimshaw voluntarily steps forward to assert her veracity, in opposition to the most incontrovertible presumptive evidence of her utter falsity.

His

His interference at all seems injudicious, but his manner of interfering still more so.

His document may appear important in his own eyes, but a few observations upon it will tend very much to lessen its importance, in the estimation of all unprejudiced persons, and the public at large.

Mr. Williams (a member of the Royal College of Surgeons, and a surgeon of his Majesty's navy) is ready to come forward with me, and assert upon oath, that the statement, in point of fact, which I have already given respecting the case of A. F., is strictly true.

A careful perusal of that, and the letter which I sent to you last month in reply to Dr. Yeats, will answer very satisfactorily the greater part of Mr. Grimshaw's letter. Nevertheless, to prove that I am not averse to discussing the merits of A. F.'s case to the fullest extent, I will gratify Mr. G. by trying to clear up the doubts which may appear to hang over the subject.

Dr. Yeats's history of her case may call for some notice in your Journal for March; the present communication will, I trust, be inserted in your next Number.

It is very true that A. F. first objected to the examination of her bladder upon the grounds of its being unnecessary and dangerous. Dr. Yeats had assured her it was so. He had even assured her and her mother that it might be the cause of her death, or, as I think she said, *her instant death*, if the catheter was introduced. Who, however, but A. F. would swear to, and who but Mr. Grimshaw would believe when sworn to, the individual words which passed in a casual conversation, and which were not noted down upon the instant? Why did A. F. report only a part of a conversation? Why does she fail to inform Mr. Grimshaw, and the public, through him, of the observations which Mr. Williams and I first made, when her objections to the use of the catheter were started? Why does not Mr. G. report that Mr. W. and I called again and again, to urge the necessity of introducing it? Dares she commit herself so much as to assert, through the medium of her friend Mr. G., that I ever *objected* to the interference or assistance of Dr. Yeats, and other or all the medical gentlemen of the town? No, she dares not and she cannot. I solemnly declare, that it was at her earnest and pathetic request alone, I delayed from day to day the calling together a general meeting of medical men (not excepting Dr. Yeats) for the express purpose of declaring my opinion of A. F., and of *insisting* upon an examination of her bladder.

Had she ever expressed a readiness to submit to the introduction troduction of the catheter, either by the advice of Dr. Yeats, or of any other medical man, Mr. Williams or I would upon the instant have sent for him, or them; but we never could for one moment obtain her permission or promise that it should be done under any circumstances. On the contrary, I have the clearest recollection that A. F. positively declared she would not, whilst she had the power of resisting, submit to any examination; and that if Dr. Y. should then, in opposition to what he had formerly advised, ever so strongly urge the introduction of the catheter, she would never agree to it. We thought it useless, under these circumstances, to request the assistance of any other medical gentlemen, until we determined to declare publicly our firm belief of the imposture.

As a corroboration of the preceding remarks, I ask, why did not A. F. or her friends, whom she so kindly entrusted with a part of our conversation respecting the use of the catheter, *at the time of its occurrence*, send for Dr. Yeats, and confront him with Williams and me?

A. F. and the persons about her, well know how urgently we advised, and even threatened her (in respect to consequences) to no purpose, whilst endeavoring to obtain her acquiescence for using the catheter.

I ask again, why she did not, *if her story is true*, request the interference of her friend Dr. Yeats during one of our many visits?

No gentleman of the medical profession could have gravely related the conversation, which is supposed to have passed, respecting the vomiting of urine. Let it, however, be remembered, that Mr. Williams had very patiently watched the effect of one emetic, a few days previous to my going for the same purpose; and that he returned from her house perfectly assured A. F. was an impostor. Let it be remembered likewise, that when I reached the house, there was half a pint of perfectly clear and healthy-looking urine ready prepared for my inspection: that I persuaded her to drink some warm water, which returned without urine, and without ipecacuanha, although this drug (I was told) had been taken to cause the vomiting. Where it could go, when the urine and warm water came up from the stomach, I know not. I might have said, that if more urine did not come up soon, I must attend to my patients. A. F. and her mother said, it was not a vomiting day. Before Mr. Williams and I expressed a determination to witness the vomiting, they did not know of "*no-vomiting* days." They talked of its coming up by pints at a time as easily as one could swallow a breakfast.

I may

I may safely leave it to the united testimony of a great proportion of the inhabitants of Bedford, to decide whether I was not perfectly correct, in regard to the amount of the charitable contributions which were levied upon their generosity. If A. F. had but enough for common exigencies, during the year preceding my calling her veracity into question, what is the state of others who are poor and sick?

I solemnly disclaim the most distant intention of unjustly assailing the moral character of a poor miserable girl.

Unfortunately, neither the prayers nor pious observations of Mr. Grimshaw avail towards clearing up the doubts that must remain upon the minds of all unprejudiced persons respecting the veracity of A. F.

Does he set the example of not intrenching upon the reputation of others? What means he by vaguely insinuating that I give painful testimony of personality? I endeavour to act, professionally, with propriety and integrity; and defy him to prove the contrary.

Analogy will avail him but little, in disproving the circumstances of suspicion that attach to the case of A. F. Where is the case, at all analogous, in its different features?

Lastly, let it be remembered that I may have occasion to make some pointed observations upon the two latter paragraphs of Mr. Grimshaw's letter which are printed in italics. The one says, "near the time of their occurrence"—the other "at the period of the occurrence itself."

*Bedford, Jan.* 11, 1814.

